# Next-generation resorbable polymer scaffolds with surface-precipitated calcium phosphate coatings

**DOI:** 10.1093/rb/rbu019

**Published:** 2015-02-12

**Authors:** Jinku Kim, Maria Hanshella R. Magno, Ophir Ortiz, Sean McBride, Aniq Darr, Joachim Kohn, Jeffrey O. Hollinger

**Affiliations:** ^1^Bone Tissue Engineering Center, Department of Biomedical Engineering, Carnegie Mellon University, Pittsburgh, PA 15219, USA, ^2^Department of Bio and Chemical Engineering, Hongik University, Sejong, Korea 339-701 and ^3^Department of Chemistry and Chemical Biology and New Jersey Center for Biomaterials, Rutgers, The State University of New Jersey, Piscataway, NJ 08854, USA

**Keywords:** tyrosine-derived polycarbonate, dicalcium phosphate dihydrate, calcium phosphate, rabbit calvarial critical size defect model, bone regeneration

## Abstract

Next-generation synthetic bone graft therapies will most likely be composed of resorbable polymers in combination with bioactive components. In this article, we continue our exploration of E1001(1k), a tyrosine-derived polycarbonate, as an orthopedic implant material. Specifically, we use E1001(1k), which is degradable, nontoxic, and osteoconductive, to fabricate porous bone regeneration scaffolds that were enhanced by two different types of calcium phosphate (CP) coatings: in one case, pure dicalcium phosphate dihydrate was precipitated on the scaffold surface and throughout its porous structure (*E1001(1k) + CP*). In the other case, bone matrix minerals (BMM) such as zinc, manganese and fluoride were co-precipitated within the dicalcium phosphate dihydrate coating (*E1001(1k) + BMM*). These scaffold compositions were compared against each other and against ChronOS (Synthes USA, West Chester, PA, USA), a clinically used bone graft substitute (BGS), which served as the positive control in our experimental design. This BGS is composed of poly(lactide co-ε-caprolactone) and beta-tricalcium phosphate. We used the established rabbit calvaria critical-sized defect model to determine bone regeneration within the defect for each of the three scaffold compositions. New bone formation was determined after 2, 4, 6, 8 and 12 weeks by micro-computerized tomography (µCT) and histology. The experimental tyrosine-derived polycarbonate, enhanced with dicalcium phosphate dihydrate, *E1001(1k) + CP*, supported significant bone formation within the defects and was superior to the same scaffold containing a mix of BMM, *E1001(1k) + BMM*. The comparison with the commercially available BGS was complicated by the large variability in bone formation observed for the laboratory preparations of E1001(1k) scaffolds. At all time points, there was a trend for *E1001(1k) + CP* to be superior to the commercial BGS. However, only at the 6-week time point did this trend reach statistical significance. Detailed analysis of the µCT data suggested an increase in bone formation from 2 through 12 weeks in implant sites treated with *E1001(1k) + CP*. At 2 and 4 weeks post-implantation, bone formation occurred at the interface where the *E1001(1k) + CP* scaffold was in contact with the bone borders of the implant site. Thereafter, during weeks 6, 8 and 12 bone formation progressed throughout the *E1001(1k) + CP* test implants. This trend was not observed with *E1001(1k) + BMM* scaffolds or the clinically used BGS. Our results suggest that *E1001(1k) + CP* should be tested further for osteoregenerative applications.

## Introduction

Osseous defects from trauma, pathological, oncological resection and developmental deformity occur in over 2 million individuals worldwide [1, 2]. In the USA, the number is estimated to be 500 000 [3]. The current ‘gold standard’ material for bone regeneration is autograft, usually harvested from the iliac crest [4–6]. Allograft is a frequent alternative to autograft while xenogeneic materials are only infrequently exploited [7].

Synthetic, bioactive bone scaffolds may provide compelling alternatives to allografts and autografts [8–11]. Currently, commercially available calcium phosphate (CP)-based bone substitutes include hydroxyapatite (HA), beta-tricalcium phosphate (β-TCP) or biphasic calcium phosphate (BCP) [1, 4]. CPs are available as granules, blocks, putties, self-setting cements and may be used as either coatings or as components in polymer/CP composites [12]. CPs may provide a bioactive stimulus for osteogenesis. Recent report suggest that ions and trace amounts of zinc, magnesium and fluoride may also support osteogenesis and thus enhance healing [13].

In a series of previous publications [15, 16, 25], we reported on the potential use of tyrosine-derived polycarbonates (TyrPC) as orthopedic implant materials. Several specific polymer compositions have been identified that exhibit excellent biocompatibility and osteoconductivity [15, 21, 25]. One of the most advanced compositions is referred to as E1001(1k). Porous bone regeneration scaffolds made of E1001(1k) and coated with dicalcium phosphate dihydrate (*E1001(1k) + CP*) were recently tested in the rabbit calvaria critical-sized defect model and found to support bone formation in the absence of exogenously added biological stimuli such as bone morphogenic protein (BMP) [16]. Based on prior reports [13], it was reasonable to assume that bone formation may be further enhanced by the inclusion of bone matrix minerals (BMM) such as zinc, manganese and fluoride within the calcium phosphate coating. The corresponding scaffolds were prepared for this study for the first time and are denoted as *E1001(1k) + BMM*.

This study has two specific aims: first, a comparison of bone regeneration in E1001(1k) scaffolds coated with either pure dicalcium phosphate dihydrate, *E1001(1k) + CP*, or with dicalcium phosphate dihydrate containing the BMM zinc, manganese and fluoride, *E1001(1k) + BMM*. Second, a comparison of these experimental bone scaffolds with a clinically used bone graft substitute (BGS).

The study design called for the comparison of three different bone scaffolds, *E1001(1k) + CP*, *E1001(1k) + BMM*, and a clinically used BGS in the rabbit calvaria critical-sized defect model. Specifically, test articles were implanted individually in a rabbit calvaria critical size defect (15 mm diameter craniotomy) and explanted at designated periods of 2, 6, 8 or 12 weeks.

## Materials and Methods

### Implant scaffold preparation

For this study, poly(DTE-co-10%DT-co-1%PEG(1k) carbonate), denoted as E1001(1k) was selected as the scaffold material. DTE stands for desaminotyrosyl-tyrosine ethyl ester, DT stands for desaminotyrosyl-tyrosine, and PEG stands for poly(ethylene glycol) with a molecular weight of 1000 g/mol. Polymer structure, nomenclature and synthetic procedures have been described in detail in a previous publication [14].

Likewise, the preparation of porous scaffolds (porosity: 85%) was described in detail previously [15]. Briefly, the scaffold fabrication procedure combines solvent casting, porogen leaching and phase separation. The final product is a highly porous material with a bimodal pore structure, consisting of micropores (<20 µm) and macropores (200–400 µm) [14].

To create a coating of precipitated calcium phosphate within the pores of the scaffold, a precipitation method was used which had been described before [16]. The porous E1001(1k) scaffolds were first immersed in 1 M CaCl_2_ solution, and then exposed to 0.96 M K_2_HPO_4_ solution. This resulted in the formation of a dicalcium phosphate dihydrate precipitate within the pores of the scaffold. These scaffolds are denoted as *E1001(1k) + CP*.

Using the same procedure, scaffolds were created that had a precipitate containing BMM. E1001(1k) scaffolds were immersed in 1 M CaCl_2_ solution containing 0.03 mM magnesium chloride and 0.01 mM zinc chloride. This was followed by exposure to 0.96 M K_2_HPO_4_ solution containing 0.01 mM of sodium fluoride. These scaffolds were denoted as *E1001(1k) + BMM*.

The % Ca by weight was determined by elemental analysis using inductively coupled plasma-optical emission spectrometry (ICP-OES, Intertek USA, NJ). Surface morphology of the scaffolds was assessed by scanning electron microscope (SEM, Amray 1830I, 20 kV).

The *E1001(1k) + CP* and *E1001(1k) + BMM* scaffolds were sterilized using ethylene oxide (EtO) (AN74i, Andersen products, Haw River, NC) and sterility was verified using a Steritest® (AN-80, Andersen Products, Haw River, NC). For comparison, a clinically used product, ChronOS, was purchased from Synthes USA.

The clinically used BGS (ChronOS), and all E1001(1k) test scaffolds were cut to identical dimensions of 15 mm diameter by 2.5 mm thickness.

### Rabbit calvarial surgery

The animal model used skeletally mature New Zealand White rabbits weighing 3.5–4.5 kg (Table 1) and a 15 mm diameter critical-sized defect as previously described [15]. Each implant scaffold was gently press fit into the single 15 mm diameter craniotomy and soft tissues were closed in layers with resorbable 4-0 Dexon sutures. Skin was closed with surgical staples. At 2, 4, 6, 8 and 12 weeks post-implantation, rabbits were euthanized humanely according to the National Institutes of Health (NIH) guidelines with an intravenous overdose of barbiturate (200 mg/kg). Harvested tissues were placed immediately into individually labeled vials of formalin at a 1:10x volume (tissue:fixative) and prepared for micro-CT and histological analysis.

**Table 1.** Treatment groups*

In-life (weeks)	TREATMENTS: E1001(1k) + CP	E1001(1k) + BMM	BGS
2	4	4	4
4	4	4	4
6	4	4	4
8	4	4	4
12	4	4	4
Total	20	20	20

*E1001(1k) is Poly(DTE-co-10%DT-co-1%PEG(1K) carbonate), where DTE, DT and PEG stands for desaminotyrosyl-tyrosine alkyl ester, desaminotyrosyl-tyrosine and poly(ethylene glycol), respectively. CP: dicalcium phosphate dihydrate, BMM: bone mineral consisting of dicalcium phosphate dihydrate in addition to Mg^2+^, Zn^2+^ and F^−^ ions.

### Micro-computed tomography

Each specimen was placed on the scanning platform of a GE eXplore Locus µCT (GE Healthcare, Piscataway, NJ) and 360 X-ray projections were collected (80 kVp; 500 mA; 26 min total scan time). Projection images were preprocessed and reconstructed into 3D volumes (20 µm resolution) on a 4PC reconstruction cluster using a modified tent-FDK cone beam algorithm (GE reconstruction software). The 3D data were processed and rendered (isosurface/maximum intensity projections) using MicroView (GE Healthcare). Trabecular bone volume in a defect site was calculated using image analysis of µCT data (MicroView, GE Healthcare). Briefly, after 3D reconstruction, each volume was scaled to Hounsfield Units (HU) using a calibration phantom containing air and water (phantom plastic); a plug within the phantom containing hydroxyapatite was used as a bone mimic for bone mineral/density calculations. Volumes were imported into Matlab (R2009b, Mathworks) for automated batch analysis [16]. Trabecular bone volume (BV) was divided by the ROI volume (total volume, TV) in order to calculate BV/TV%.

### Histology and histomorphometry

The harvested samples were dehydrated in ascending grades of ethanol, cleared in xylene at 4°C to minimize implant solvation during the processing and embedded in poly(methyl methacrylate). The specimens were cut and ground to 30 µm thick sections with an Exakt diamond band saw and MicroGrinder (Exakt Technologies, Oklahoma City, OK). The histology slides were stained with Sanderson’s Rapid Bone Stain and counterstained with van Gieson’s picrofuchsin, which resulted in soft tissue staining blue and bone staining pink/red.

The coronal plane of the specimens were stained with Sanderson’s Rapid Bone Stain and counterstained with van Gieson’s picrofuchsin (×1.5 magnification).

New bone formation was measured by an image analysis program (Optimas version 6.5, Media Cybernetics, Bethesda, MD). Briefly, the defect area (region of interest, ROI) on each histology section (×1.5) was selected and the areas of new bone were determined based on predetermined color thresholds. The percentage of new bone area was obtained by dividing the bone area by whole defect area.

### Statistics

All data were reported as an arithmetic mean ± standard deviation of four replicates (*n* = 4) and tested for significance at *P* < 0.05 using single factor analysis of variance (ANOVA) and Tukey *post-hoc* test.

## Results

### Scaffold preparation and characterization

**Figure 1.**
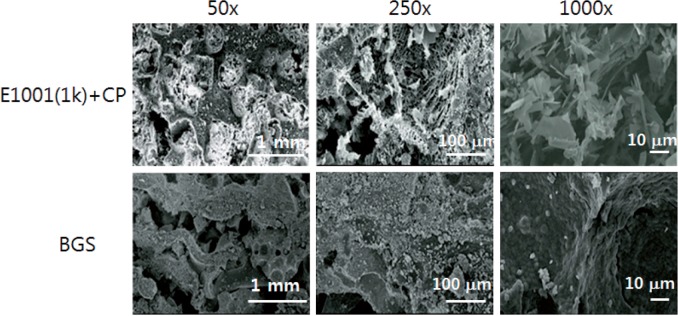
Representative SEM images of *E1001(1k) + CP* scaffolds and ChronOS, a commercially available BGS at different magnifications of ×50, ×250 and ×1000.

SEM images (Fig. 1) suggested that *E1001(1k) + CP* scaffolds had macro- and micropores throughout the entire volume of the scaffold. As reported before, the pore sizes were <20 µm for the micropores and between 200 µm and 400 µm for the macropores. No changes in pore architecture were noted when different areas of the scaffold were examined. These findings indicate that the fabrication procedure yielded similar scaffold architectures as compared to previous results [16].

Precipitated dicalcium phosphate dihydrate was present distributed throughout all pores and on the scaffold surface. Compared to the E1001(1k) + CP scaffolds, the control BGS had a less regular architecture with irregularly shaped micropores and far less macropores than the E1001(1k) + CP scaffolds. While the detailed analysis of the differences between the clinically used BGS device and E1001(1k) + CP scaffolds is beyond the scope of the publication, it was obvious that there were significant architectural differences between these two scaffold types (Fig. 1).

### Rabbit surgeries and necropsies

**Figure 2.**
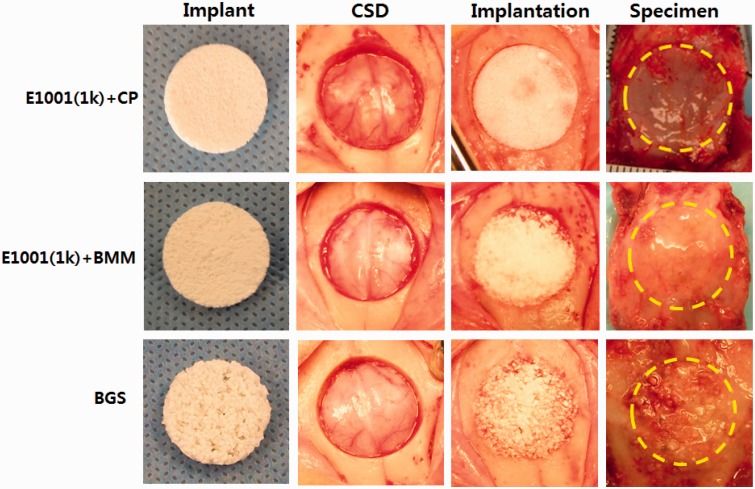
Implants and surgery images. All test implants (scaffolds) fit snugly into the craniotomy defects. There were no adverse tissue observations (e.g., necrosis) at necropsy. Implant: appearance of the implant prior to use. CSD: critical-size defect generated by drilling a 15-mm wide hole into the skull bone. Implantation: surgery site immediately after fitting a test implant into the defect. Specimen: appearance of the implant site (outlined by the dotted yellow line) after the animal was sacrificed and the implant site with its surrounding bone was removed for tissue processing (necropsy specimen).

At surgery, the E1001(1k)-based scaffolds were pliable and retained their shape during implantation. The commercially available BGS strips were particulate and brittle and required more careful handling during insertion (Fig. 2). Moreover, E1001(1k)-based scaffolds imbibed blood, which may have a positive impact on wound healing due to the accumulation of endogenous osteogenic and angiogenic cues [17, 18] (Fig. 2).

### Micro-CT analysis

**Figure 3.**
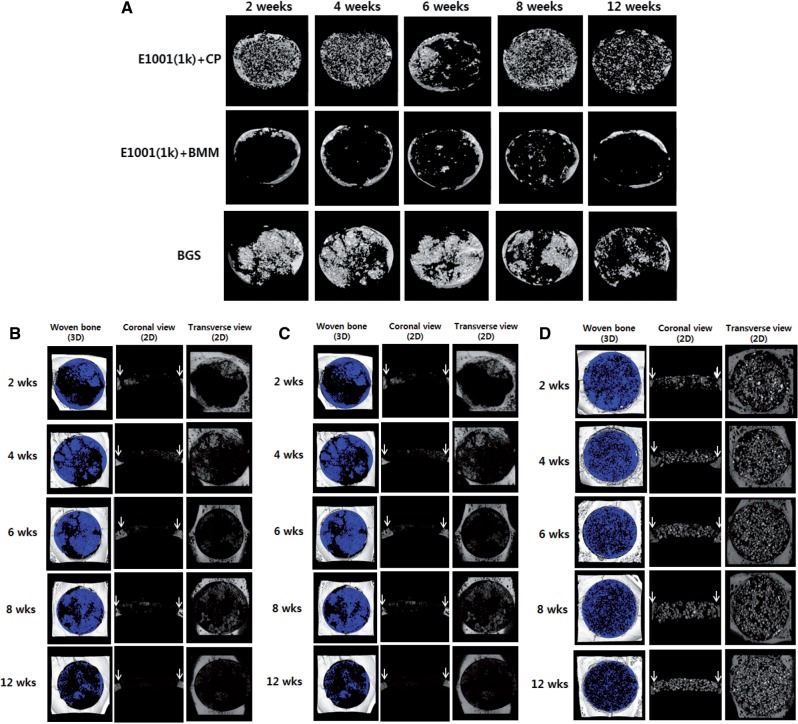
Representative µCT images for implant sites in the rabbit calvarial critical-size defect model at 2, 4, 6, 8 and 12 weeks. Blue color in the 3D reconstruction images of the defects indicates trabecular bone. White arrows in the 2D coronal images indicate the defect margin. Remaining β-TCP fragments (bright white spots) can be seen in the 2D coronal and transverse images of the BGS scaffolds. Note that the raw data shown for BGS are misleading: Most of the bright spots in BGS were residual calcium phosphate and not newly grown bone. (A) Snapshots of 3D reconstruction implant sites treated with *E1001(1k) + CP*, *E1001(1k) + BMM* and BGS (ChronOS). (B) Detailed µCT images of the implant sites treated with *E1001(1k) + CP.* (C) Detailed µCT images of the implant sites treated with *E1001(1k) + BMM.* (D) Detailed µCT images of the implant sites treated with BGS (ChronOS).

The µCT 2D images and 3D renderings suggested a difference in bone regeneration among treatment groups. The detailed µCT images with various anatomic directions were also shown in Fig. 3B–D. The *E1001(1k) + CP* treated group had marginal new bone formation at 2 and 4 weeks. Bone regeneration increased at 6, 8 and 12 weeks (Fig. 3A). In contrast, the *E1001(1k) + BMM* scaffolds did not show significant bone formation, even at 12 weeks.

**Figure 4.**
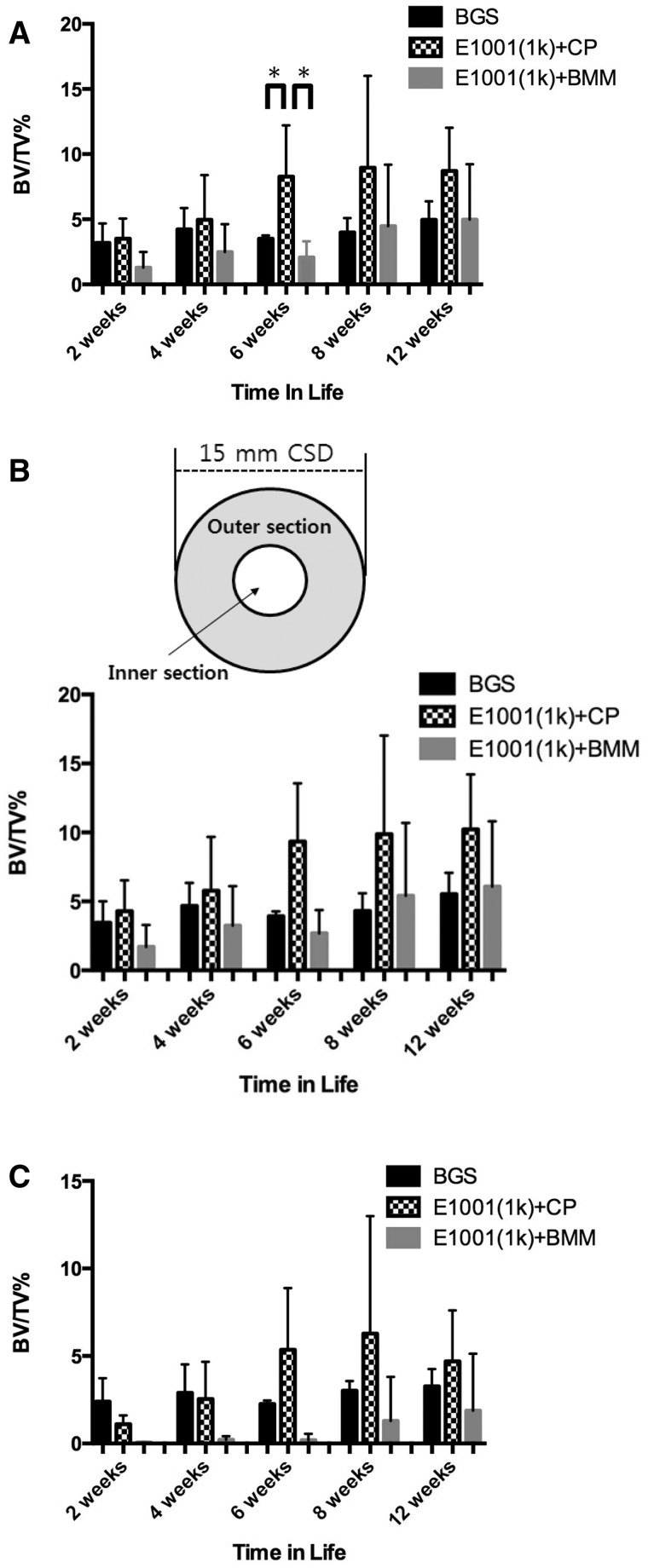
Quantitative analysis of bone regeneration in the defect sites at 2, 4, 6, 8 and 12 weeks. Data are reported as a mean ± standard deviation for *n* = 4. *Represents a statistically significant difference (*P* < 0.05) between two groups. BV stands for trabecular bone volume within the defect. TV stands for the total defect volume. (A) Whole bone volume in the critical size defect expressed as mm^3^ of new bone in the defect. (B) Segmented bone volume in the outer section only (donut-like, shaded area). Here, the total volume (TV) represents the volume of the outer section only. (C) Segmented bone volume in the inner section of the critical size defect only, determined by µCT image analysis. The inner section is the donut hole section (small circle) in the critical size defect. Here the total volume () represents the volume of the inner section only.

Although the control BGS appeared to show greater white regions in the defects (Fig. 3A), these white regions turned out to be calcium phosphate from the BGS after µCT analysis (actual bone can be differentiated from calcium phosphate due to the differences in predefined Hounsfield Unit thresholds). Thus, the control BGS implants appeared to form negligible amounts of bone throughout the defect area. Substantial amount of beta-TCP fragments persisted in the defects throughout all time points, indicating minimal scaffold degradation over time. Total bone volume data suggested that *E1001(1k) + CP*-treated defects had significantly more new bone volume than BGS-treated groups at 6 weeks (Fig. 4A).

We noticed a number of experimental difficulties during data analysis. First, the E1001(1k) + CP scaffolds are individually prepared laboratory specimens for which slight variations in manufacturing can lead to noticeable variations in their performance during *in vivo* testing. It is therefore not surprising that the E1001(1k) + CP scaffold exhibited larger variability in their performance as reflected by their significantly greater error bars. Second, in micro-CT and histomorphometic analysis, it is sometimes difficult to determine the exact ‘region of interest’ (ROI) around the bone–scaffold interface. Since bone is densest at this interface, even small errors in determining the ROI can significantly affect the results.

To ameliorate these problems, we separated the total defect site into a donut-shaped outer region that included the critical bone–implant interface and an inner region. We hoped that by looking at these two regions separately, the variability of our results could be reduced. The results for the donut-shaped outer layer and the central inner layer are presented in Fig. 4B and C, respectively. Specifically, the inner section had a diameter of 7 mm, leaving a donut-shaped outer section with a width of 3.5 mm on each side (shaded area in the insert in Fig. 4B).

Although this way of presenting the data did not reduce the variability of the results, we did gain an important insight from this analysis: The data showed that although all three treatment groups had new bone formation in the defect, only the *E1001(1k) + CP* scaffolds achieved a normalized bone volume (BV/TV) above 5% in the inner section of the defect (Fig. 4B).

### Histological analysis and histomorphometry

Qualitative analysis of histology suggested trends similar to the µCT data. The *E1001(1k) + CP* scaffolds had regenerated bone in the middle of the defect area by 6 weeks. This was a unique phenomenon, since *TyrPC + BMM* and BGS (ChronOS) regenerated modest amounts of bone only at the periphery of the defect, where bone and implant were in direct apposition.

As a general observation that is valid for all three types of tested scaffolds, bone regeneration was slow, with little new bone formation at 2 and 4 weeks and evidence for some fibrous connective tissues being formed within the defect sites. New bone formation was predominantly evident at the margin of the host bone and alongside dural area in the defects at 6 and 12 weeks, which confirmed the µCT data.

The three treatment groups, *E1001(1k) + CP*, *E1001(1k) + BMM* and BGS (ChronOS) were biocompatible and appeared to have a compatible host bone–implant interface with neither connective tissue nor inflammatory exudate between the host bone and the implants.

**Figure 5.**
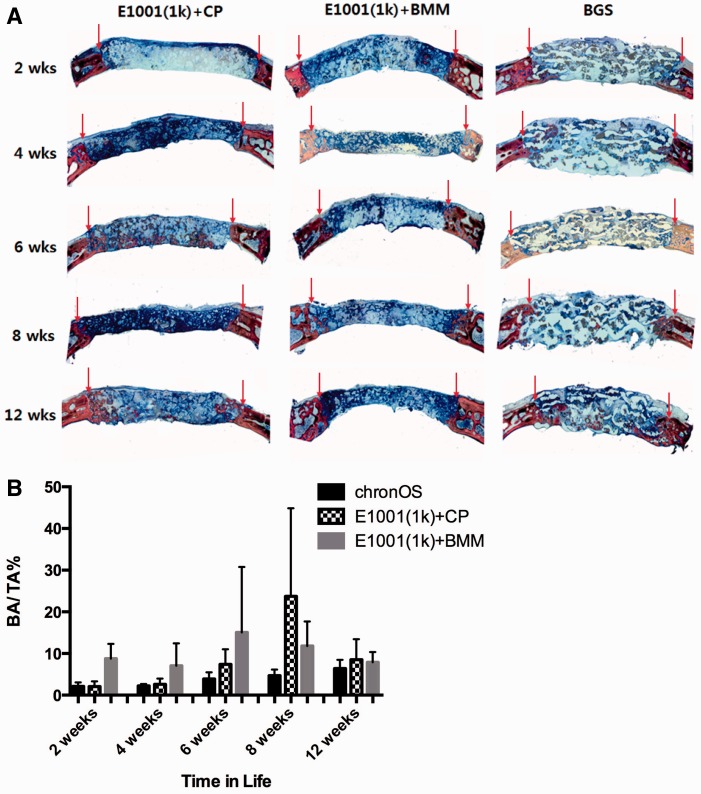
Histology and histomorphometry. (A) Representative histomicrographs (×1.5; coronal plane) at the designated time periods treated with *E1001(1k) + CP*, *E1001(1k) + BMM* and BGS (ChronOS). Stain: Sanderson’s Rapid Bone Stain, counterstained with van Gieson’s picrofuchsin. Red arrows in the 2D coronal images indicate the defect margin. (B) New bone area (%) in the defect site determined by histomorphometry. BA: bone area; TA: total area of the implant site. Data are reported as a mean ± standard deviation for *n* = 4. *Represents a statistically significant difference (*P* < 0.05) between two groups.

Histomorphometry data suggested a trend toward increased bone formation in the *E1001(1k) + CP*-treated implant sites at 8 and 12 weeks. This is similar to the micro-CT data. However, because of the large variation in bone formation between the individual *E1001(1k) + CP* treated implant sites, there were no significant histomorphometic differences between *E1001(1k) + CP* and BGS (ChronOS) treated sites (Fig. 5B).

Finally, a somewhat surprising and counter-intuitive result was our finding that adding BMM to the dicalcium phosphate dihydrate coatings did not improve bone regeneration in E1001(1k) scaffolds.

## Discussion

In principle, totally synthetic bone regeneration scaffolds are a very attractive alternative to the use of autografts or allografts, but they clearly lack all biological signaling cues and therefore tend to perform poorly in the clinic unless augmented with bone marrow aspirate, BMP, or other biological components. It has been a significant research challenge to identify an engineering approach for bone regeneration scaffolds that can approach the regenerative performance of autografts. The current study is part of this continuing effort to address this challenge.

*E1001(1k) + BMM* (e.g., E1001(1k) scaffolds enhanced with a mix of BMM) contained dicalcium phosphate dihydrate in addition to magnesium, zinc and fluoride. Reports suggest that this composition may change crystal morphology, dissolution, osteoclast activity and proteins involved in bone mineral production [19]. In our study, this BMM composition did not improve bone regeneration as compared to *E1001(1k) + CP* (e.g., E1001(1k) scaffolds enhanced with a precipitate of dicalcium phosphate dihydrate only). We recognize that the development of an effective BMM composite will require extensive optimization of the type and concentration of BMM used. Therefore, our results should only be regarded as a first preliminary indication that a more detailed investigation is needed to assess the potential of various bone mineral mixtures to enhance bone regeneration in combination with E1001(1k) scaffolds.

To facilitate a comparison of our experimental scaffolds with a clinically used BGS, we included ChronOS as a positive control in our experimental design.

*E1001(1k) + CP* scaffolds were effective in regenerating bone. Even in the absence of optimized manufacturing procedures, these *E1001(1k) + CP* scaffolds seem to have a tendency to perform better than the clinically used control BGS. However, because of the lack of optimized manufacturing procedures, E1001(1k) + CP scaffolds exhibited high variability which made it impossible to obtain statistically significant differences between *E1001(1k) + CP* and the control BGS in this small study.

However, there were interesting differences in the performance between *E1001(1k) + CP* and the control BGS. µCT and histology showed an increase in bone regeneration at the later time points only in defects treated with *E1001(1k) + CP*. Possible explanations for the increased bone formation in *E1001(1k) + CP* scaffolds relative to control BGS at these later time points include: (i) *E1001(1k) + CP* scaffolds may recruit osteogenic cells due to their hydrophobic surface properties [20–22], or (ii) free carboxylic acid groups present on the surface of TyrPC scaffolds may act as nucleation sites for the formation of hydroxyapatite [23, 24].

Scaffold architecture will also affect bone regeneration. E1001(1k) scaffolds have macropores in the range of 200–400 µm. This pore range has been reported as optimal for bone regeneration [25, 26]. In addition, our previous reports demonstrated the presence of micropores less than 20 μm in the scaffolds [14, 27]. The control BGS had a very different pore architecture. Another architectural difference is that the dicalcium phosphate dihydrate precipitate on the E1001(1k) + CP scaffolds was distributed throughout the scaffold in the form of a surface coating, while the control BGS is a composite of beta-TCP particles embedded within a polymer matrix [28].

Histology data suggested a trend similar to the µCT data. Osteoconduction and osteointegration were evident for the *E1001(1k) + CP* scaffolds at 6 weeks. The two other treatment groups (*E1001(1k) + BMM* and BGS) had marginal bone formation. Notable macroscopic histological difference among treatment groups was the existence of substantial void regions throughout the defects treated with the BSG (Fig. 5A). This phenomena was observed in the µCT images as well (2D coronal plane of the BSG in Fig. 3B). The observation may be a consequence of *in situ* swelling of the BGS implants [29]. Swelling of synthetic bone substitutes may have detrimental effects and may hinder tissue regeneration due to the collapse of the implant upon degradation [29, 30].

## Conclusions

This study provided a comparison of the bone regeneration potential of three different scaffold compositions in the widely used critical defect rabbit calvaria model. Our studies showed that all three tested scaffold compositions were biocompatible and did not elicit a clinically significant inflammatory response at the implant site.

The size of the error bars in Figs 4 and 5 clearly demonstrates the need for careful control of the manufacturing process: the clinically used BGS is produced in a commercial manufacturing process and has consistently the least variability in its *in vivo* performance. In contrast, the experimental E1001(1k) scaffolds are produced in the laboratory and show substantial variability in their *in vivo* performance. Although this variability impacted our ability to obtain statistical significance in our comparative data, we can reach the following qualitative conclusions:

1. Scaffolds containing BMM were the poorest performers at all time points.
2. When we divided the ROI into an outer section and an inner section, it is evident that osteoconduction in the *E1001(1k) + CP* group progressed durally and that among the three tested scaffolds, only *E1001(1k) + CP* scaffolds regenerated more that 5% bone volume within the inner section of the defect.
3. The differences between *E1001(1k) + CP* scaffolds and the clinically used BGS are less pronounced. While *E1001(1k) + CP* scaffolds have a tendency to perform better that the BGS control, this trend reached statistical significance only at the 6-week time point.

Overall, the *E1001(1k) + CP* scaffolds appear to be suitable biomaterials for clinical bone graft procedures. However, significant additional studies must be completed to validate clinical opportunities.
